# Elevated monocyte to high-density lipoprotein cholesterol ratio correlates with clinical severity in acute inflammatory demyelinating polyradiculoneuropathy patients

**DOI:** 10.3389/fneur.2022.955933

**Published:** 2022-09-27

**Authors:** You-Fan Peng, Miao Luo, Qing-Song Zhang

**Affiliations:** ^1^Department of Respiratory and Critical Care Medicine, Affiliated Hospital of Youjiang Medical University for Nationalities, Baise, China; ^2^Life Science and Clinical Research Center, Affiliated Hospital of Youjiang Medical University for Nationalities, Baise, China

**Keywords:** Guillain-Barré syndrome, monocyte to high-density lipoprotein cholesterol ratio, acute inflammatory demyelinating polyradiculoneuropathy, acute motor axonal neuropathy, acute motor sensory axonal neuropathy

## Abstract

While monocyte to high-density lipoprotein cholesterol ratio (MHR) has been reported to be associated with nervous system lesions, the role of MHR has not been determined in patients with Guillain-Barré Syndrome (GBS). The purpose of our study was to explore the role of MHR in patients with GBS. A total of 52 GBS patients were involved in the study retrospectively, including patients with acute inflammatory demyelinating polyradiculoneuropathy (AIDP), acute motor axonal neuropathy (AMAN), and acute motor sensory axonal neuropathy (AMSAN). We used Hughes Functional Grading Scale (HFGS) score to evaluate functional status in GBS patients. Among patients with different subtypes of GBS, MHR was significantly elevated in those with demyelination compared to patients without demyelination (*p* < 0.001); AIDP patients had an increased MHR compared with AMAN or AMSAN patients (*p* = 0.001; *p* = 0.013). There was a positive correlation between MHR and HFGS score (r = 0.463, *p* = 0.006) in AIDP patients, but not in AMAN or AMSAN. Multiple linear regression analysis revealed that MHR was independently associated with HFGS score (beta = 0.405, *p* = 0.013) in AIDP patients. Our study suggests that MHR as an inflammatory marker is elevated in patients with AIDP compared to AMAN or AMSAN patients, while MHR has a positive correlation with clinical severity in AIDP patients, suggesting that MHR may provide an additional information to reflect the pathophysiology of AIDP.

## Introduction

Guillain-Barré Syndrome (GBS) is an acute inflammatory and immune-mediated peripheral nervous disease (1). Clinically, clinical symptoms, electrophysiological features, and cerebrospinal fluid test have been used to diagnose and evaluate GBS patients (1). At present, some biological markers have certain clinical value for patients with GBS (2). Monocytes have been involved in major chronic inflammatory diseases (3). High-density lipoprotein cholesterol (HDL-C) has been regarded as a biomarker of cardiovascular disease risk (4). However, HDL-C has been found to be associated with inflammatory conditions (5). Recently, there is evidence to suggest that the monocyte to high-density lipoprotein cholesterol ratio (MHR), as a calculated value, is associated with multiple sclerosis (6). In addition, MHR is significantly increased in migraine patients compared with controls (7). Increased MHR values have been related to polyneuropathy in diabetic patients (8). Thus, we speculate that MHR may be associated with nervous system lesions; we aimed to assess the clinical role of MHR in patients with GBS.

## Materials and methods

Our study included 52 patients with GBS retrospectively. All patients were diagnosed according to the diagnostic criteria of GBS (9). All patient's conditions were assessed on admission to hospital. For the admission blood examinations, blood samples were collected for a blood routine test and biochemical test, and a further cerebrospinal fluid test and electrophysiological examination were used to estimate all GBS patients. The time of blood sample collection was performed on admission for all patients with GBS, thus, it was consistent for all patients. Patients with AIDP and AMAN were determined according to the criteria set out by Ho et al. (10); patients with AMSAN were determined based on the electrophysiological findings (11). All GBS patients were divided into three subtypes: 34 patients with acute inflammatory demyelinating polyradiculoneuropathy (AIDP), 7 patients with acute motor axonal neuropathy (AMAN), and 11 patients with acute motor sensory axonal neuropathy (AMSAN).

Patients were excluded from the study if they had cardiovascular disease, renal insufficiency, mononucleosis, active tuberculosis, migraine, rheumatic disease, or malignancies. The functional status was estimated by Hughes Functional Grading Scale (HFGS) score in patients with GBS, which is a widely recognized scoring system to evaluate the functional status of GBS patients (12). The study was approved by the Ethics Committee of Affiliated Hospital of Youjiang Medical University for Nationalities, and was conducted in accordance with the Declaration of Helsinki.

### Statistical analysis

The continuous variables are expressed as the means ± SD for normal distribution and median (interquartile range) for non-normal distribution. Categorical variables are expressed as a percentage. Categorical variables were compared with Chi-square test or Fisher's exact test. When appropriate, continuous variables between two groups were compared by Student t test or Mann-Whitney U test, and continuous variables for three groups were compared by ANOVA or Kruskal-Wallis H-test with *post hoc* analysis. The bivariate correlation was assessed by Pearson or Spearman approach appropriately. Multiple linear regression analysis was performed to determine variables that could be independently associated with MHR in AIDP patients. The statistical analyses were carried out by SPSS version 25.0 statistical software. Statistical significance was defined as a *p* < 0.05.

## Results

### The associations between MHR and time from onset to admission in different subtypes of GBS patients

To estimate whether the timing of blood specimen collection used to calculate the MHR after onset may have an influence on MHR that reflects the disease status of GBS patients, the correlations between MHR and time from onset to admission (TOA) were assessed in different subtypes of GBS patients, and the values of TOA were compared in different subtypes of GBS patients. The results found that MHR was not correlated with TOA in all GBS patients, and there were no correlations between MHR and TOA in different subtypes of GBS patients. TOA had no significant difference between demyelinating patients (AIDP) and patients without demyelination (AMAN and AMSAN), and there were no significant differences in TOA between different subtypes of GBS patients (Table 1).

**Table 1:** The main characteristics in patients with different subtypes of GBS.

**Variable**	**AIDP**	**AMAN/ AMSAN**	***p* value**
Male sex, n (%)	20 (58.8)	8 (44.4)	0.322
Age (years)	53 (40–63)	49 (40–54)	0.236
Time from onset to admission (Days)	9 (5–14)	7 (5–10)	0.580
Hypertension, n (%)	5 (14.7)	1 (5.6)	0.651
Diabetes, n (%)	4 (11.8)	1 (5.6)	0.648
Hyperlipemia, n (%)	22 (64.7)	8 (44.4)	0.159
HFGS score	3.0 (1–4)	2.5 (1–3)	0.295
Neutrophil counts (10^9^/L)	4.34 ± 1.63	3.24 ± 1.13	0.014
Monocyte counts (10^9^/L)	0.54 (0.46–0.64)	0.35 (0.30–0.47)	0.001
Platelet counts (10^9^/L)	260.21 ± 82.56	231.44 ± 67.11	0.210
Total cholesterol (mmol/L)	4.03 ± 1.07	4.32 ± 1.18	0.369
Triglyceride (mmol/L)	1.25 (0.90–1.84)	1.18 (0.86–1.65)	0.729
High-density lipoprotein cholesterol (mmol/L)	1.05 ± 0.24	1.35 ± 0.46	0.017
Low-density lipoprotein cholesterol (mmol/L)	2.62 ± 0.90	2.67 ± 0.96	0.866
Alanine aminotransferase(U/L)	25 (16–39)	16 (11–24)	0.087
Aspartate aminotransferase(U/L)	21 (18–31)	20 (16–26)	0.847
Monocyte count to high-density lipoprotein cholesterol ratio	0.55 (0.44–0.67)	0.26 (0.22–0.37)	<0.001

### The associations between MHR and different subtypes in patients with GBS

The main characteristics in patients with different subtypes of GBS are shown in Table 1. We compared the MHR values in different GBS subtypes; the values of MHR were significantly higher in patients with demyelination (AIDP) than patients without demyelination (AMAN and AMSAN) (*p* < 0.001). We also found that the values of MHR were significantly increased in AIDP patients compared to AMAN or AMSAN patients (*p* = 0.001; *p* = 0.013), however, no significant difference was observed in MHR values between AMAN and AMSAN in GBS patients.

### The correlations between MHR and HFGS score in different subtypes of GBS patients

Further, to explore the association between MHR and clinical severity in patients with GBS, the correlations between MHR and HFGS score were assessed in different subtypes of GBS; the results found that MHR was positively correlated with HFGS score (r=0.463, *p* = 0.006) in patients with AIDP. However, no significant correlation between MHR and HFGS score was found in patients with AMAN or AMSAN.

### Multiple linear regression analysis for factors associated with MHR in patients with AIDP

The multiple linear regression analysis was conducted to estimate which factors were independently associated with MHR in patients with AIDP. Age, hypertension, diabetes, hyperlipemia, TOA, and HFGS score served as independent variables. At the multiple linear regression analysis, the results found that HFGS score was one of the independent determinants of MHR (beta = 0.405, *p* = 0.013) in AIDP patients, and hyperlipemia was also a significant contributing factor to MHR (beta=0.415, *p* = 0.012) in AIDP patients, as shown in Table 2.

**Table 2:** The multiple linear regression analysis showing the correlation between MHR and HFGS score in patients with AIDP.

**Variables**	**Standardized coefficients Beta**	**_t_**	***p* value**
Age	0.027	0.147	0.885
Hypertension	−0.029	−0.159	0.875
Diabetes	−0.001	−0.005	0.996
Hyperlipemia	0.415	2.692	0.012
Time from onset to admission	0.110	0.686	0.499
HFGS score	0.405	2.644	0.013

## Discussion

Our study implicated the role of MHR in patients with GBS, and revealed the association between MHR and different subtypes of GBS. The present report found that the values of MHR were significantly increased in AIDP patients compared with AMAN and AMSAN patients, and demonstrated a positive correlation between MHR and HFGS score in patients with AIDP.

The main lesions of AIDP are the segmental demyelination of nerve; only the axons of motor neurons are damaged in AMAN, whereas the axons of both motor and sensory neurons are damaged in AMSAN (13). Our study found that MHR was elevated in AIDP patients compared with AMAN and AMSAN patients; moreover, MHR was positively correlated with HFGS score in patients with AIDP, but not in AMAN or AMSAN. Considering the timing of blood sample collection after onset may affect that MHR reflects the disease status of GBS patients, we further investigated the correlations between MHR and TOA in different subtypes of GBS patients, indicating that TOA had no influence on MHR values. Monocytes in circulation populate tissues as macrophages during inflammation. Monocyte-derived macrophages are effector cells in neuroinflammatory and neurodegenerative disorders (14, 15). Evidence has been provided that peripheral inflammation is associated with the progression of demyelinating diseases (16). It has been reported that nerve-specific T-cells, macrophage scavenging, and complement activation are involved with the demyelination of AIDP (17). Importantly, the infiltration of inflammatory mononuclear cell is observed in GBS patients characterized by myelin multifocal loss of the peripheral nervous system with relative preservation of axons, as well as more leucocytes and T cells are found in the endoneurium than control cases (18). To sum up, these reports indicate that cell-mediated neuroinflammation may be associated with demyelinating lesion in AIDP patients. It has been highlighted that the axonal patterns of GBS have been associated with Campylobacter jejuni infection with autoimmune attack of gangliosides, and the axon is obviously destroyed without inflammatory infiltration (19, 20). Neutrophil to lymphocyte ratio and platelet to lymphocyte ratio have been considered as inflammatory markers in inflammatory disease (21). Ozdemir HH (22) attested that elevated pretreatment neutrophil to lymphocyte ratio and platelet to lymphocyte ratio are significantly associated with AIDP, suggesting cell-mediated neuroinflammation may be a main characteristic in AIDP, rather than in AMAN or AMSAN.

It has been reported that elevated monocyte counts are positively associated with disease severity in GBS patients (23). Our study found that MHR was correlated with functional status in AIDP patients. MHR as a combined parameter is a calculated value based on monocyte counts and HDL-C, thus, MHR may be a more stable way to assess disease status than a single laboratory parameter. High monocyte counts and low HDL-C levels have been reported in inflammatory disorders (5, 24). Monocytes are important cells for the secretion of pro-inflammatory and pro-oxidant cytokines in inflammatory conditions (25). A previous study observed that anti-inflammatory treatment decreases peripheral blood monocyte counts in patients with inflammatory bowel disease (26). It has been shown that higher monocyte counts as an inflammatory marker are related to the occlusion of small vessels in patients with hypertension (27).

High sensitivity C-reactive protein (hs-CRP) levels have been found to be increased in patients with low HDL-C concentrations (28). HDL-C has been reported to be inversely associated with systemic inflammation (29), and serum HDL-C levels have been attested to be inversely correlated with hs-CRP and tumor necrosis factor-α in non-diabetic hemodialysis patients (30). Indeed, MHR has been regarded as an indicator of inflammation to reflect systemic inflammatory response (31), and MHR has been associated with nervous system disease mediated by inflammation (6–8). Therefore, it is predictable that cell-mediated neuroinflammation with demyelinating lesions may increase MHR values in patients with AIDP, and that MHR as an inflammatory marker may reflect the lesions of cell-mediated neuroinflammation in patients with AIDP.

Certain limitations should be taken into account in this study. First, the sample size is small in our study, especially when the subtypes of GBS are considered in analysis, which may limit the extrapolation ability of the results. Second, the effects of diet and lipid-lowering agents on HDL-C levels have not been evaluated in patients with GBS, which may be confounding factors that cannot be ignored. Third, a direct association between MHR and indicators of cell-mediated neuroinflammation has not been assessed in patients with AIDP. Fourth, the present study did not observe whether MHR also decreased with symptom recovery in GBS, especially for AIDP. Fifth, the relationship between elevated MHR and poor outcomes has not been evaluated in GBS patients.

## Conclusion

Overall, MHR as an inflammatory marker is increased in patients with AIDP compared with AMAN or AMSAN patients, and MHR is positively correlated with clinical severity in AIDP patients, indicating that MHR may give an additional information to reflect the pathophysiology of AIDP. However, a large sample study is needed to confirm our current results.

## Data availability statement

The original contributions presented in the study are included in the article/supplementary material, further inquiries can be directed to the corresponding author.

## Ethics statement

The studies involving human participants were reviewed and approved by Ethics Committee of Affiliated Hospital of Youjiang Medical University for Nationalities. Written informed consent from the participants' legal guardian/next of kin was not required to participate in this study in accordance with the national legislation and the institutional requirements.

## Author contributions

Y-FP conceived and designed the study. Y-FP performed the data analysis and wrote the manuscript. Y-FP, ML, and Q-SZ collected the data. All authors contributed to the article and approved the submitted version.

## Conflict of interest

The authors declare that the research was conducted in the absence of any commercial or financial relationships that could be construed as a potential conflict of interest.

## Publisher's note

All claims expressed in this article are solely those of the authors and do not necessarily represent those of their affiliated organizations, or those of the publisher, the editors and the reviewers. Any product that may be evaluated in this article, or claim that may be made by its manufacturer, is not guaranteed or endorsed by the publisher.
